# A Healthy Pregnancy During Treatment of Metastatic Melanoma with Immune Checkpoint Inhibitors: A Case Report

**DOI:** 10.3390/jcm14238591

**Published:** 2025-12-04

**Authors:** Corinna Schneider, Melánia Pozsgai, Csongor Németh, Zita Battyáni, Zsuzsanna Lengyel

**Affiliations:** Department of Dermatology, Venerology and Oncodermatology, University of Pécs, 7632 Pécs, Hungary

**Keywords:** dermatology, melanoma, immune checkpoint inhibitors, PD-1 inhibitors, anti-CTLA4 inhibitors, pregnancy

## Abstract

**Background/Objectives**: With the increasing use of immune checkpoint inhibitors (ICIs), their administration in pregnant patients is expected to become more frequent. Although immunotherapy has transformed melanoma treatment, its use during pregnancy remains complex and controversial. **Methods**: We present the case of a young female patient diagnosed with advanced melanoma in whom combination ICI therapy was initiated. **Results**: During maintenance nivolumab treatment, a routine staging CT scan revealed an incidental pregnancy. Immunotherapy was discontinued upon pregnancy detection. The pregnancy proceeded without significant complications related to melanoma or the immunotherapy. During follow-up, the patient remained in remission and delivered a healthy male infant at 38 weeks of gestation. **Conclusions**: The most frequently reported side effects of ICIs during in utero exposure include fetal growth restriction, premature delivery, fetal distress syndrome, and occasional congenital abnormalities such as hypothyroidism and hand malformations. While existing evidence highlights potential risks, isolated case reports—including the present case—demonstrate that favorable pregnancy and neonatal outcomes are possible with careful monitoring and multidisciplinary care. Given the limited literature on ICI use during pregnancy, our case adds meaningful clinical insights to the field and underscores the need for further research and data collection to establish definitive guidelines, with an emphasis on individualized risk assessment and multidisciplinary care.

## 1. Introduction

Immune checkpoint inhibitors (ICIs) have been identified as a major advancement in cancer treatment, with their capacity to target the interaction between programmed cell death 1 (PD1) and programmed cell death ligand 1 (PD-L1), as well as their ability to block cytotoxic T lymphocyte antigen 4 (CTLA-4), resulting in enhanced precision and efficacy in treatment. The restoration of T-cell–mediated antitumour activity, which had previously been suppressed, is achieved by the blocking of these key inhibitory pathways.

It is evident that agents such as pembrolizumab, nivolumab, and ipilimumab have been continuously demonstrating durable responses and improved survival in cancers including melanoma, non-small cell lung cancer, and renal cell carcinoma.

The clinical benefit and safety profiles of these therapies have been extensively evaluated across multiple clinical trials [1]. However, challenging populations such as pregnant patients, patients with autoimmune diseases or viral infections, and patients who have undergone solid organ transplants are excluded from clinical trials. This results in a paucity of data on which to base decisions regarding the treatment of these patient populations with ICIs [2]. It is important to note that current guideline recommendations (ESMO, NCCN) advise avoiding conception during ICI therapy and for an extended period thereafter: at least 5 months after the last dose of ipilimumab and at least 3 months after the final dose of nivolumab monotherapy. The utilization of active contraception is strongly recommended during this specified interval [3,4]. Treating cancer during pregnancy presents a complex challenge, requiring a careful balance between managing disease progression and minimizing risks to both the mother and the fetus.

In this article, the authors present the case of a young female patient who was diagnosed with metastatic melanoma and, while receiving maintenance nivolumab therapy, conceived and gave birth to a healthy child. In addition, we aim to provide a summary of data from the last 10 years relating to the administration of ICIs during pregnancy.

## 2. Case Report

**Figure 1:**
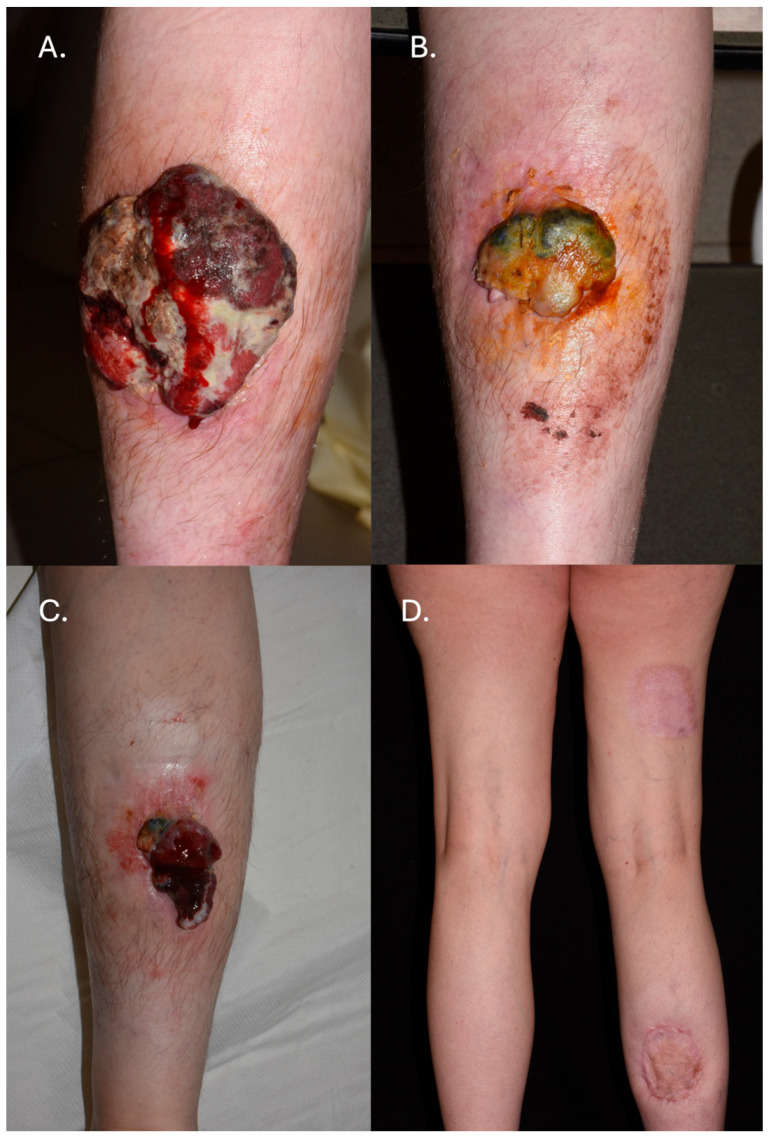
The primary melanoma at the time of the patient’s initial presentation in December 2019 (**A**); regression of primary melanoma at 6 months of dabrafenib and trametinib treatment (June 2020). At this time, pulmonary metastases were detected on CT scan (**B**); tumor size 8 months after starting ipilimumab and nivolumab therapy (**C**); after surgical removal of the tumor (**D**).

A 37-year-old woman, who had given birth to two children and had no family history of malignant skin cancers, initially presented at our clinic—Department of Dermatology, University of Pécs—in October 2019 with an exophytic, large, egg-sized mass located on her right lower leg with palpable enlarged right inguinal lymph nodes (Figure 1A).

Biopsies taken from both the primary tumor and the lymph node confirmed the diagnosis of malignant melanoma. CT scans (brain, chest, abdomen) were performed, revealing lymphadenomegaly in the right inguinal lymph nodes and a subpleural nodule requiring further follow-up. Molecular testing identified the presence of the BRAF V600E mutation. According to a PET-CT scan conducted in November 2019, aside from metabolic activity in the malignant tumor on the right lower limb and lymph nodes, no FDG uptake was detected elsewhere. Given the unresectability of the primary tumor and the marked enlargement of the inguinal lymph node conglomerate, we confirmed an unresectable Stage III melanoma. The multidisciplinary tumor board recommended initiating systemic therapy. As a result, and in agreement with the financial reimbursement, treatment with dabrafenib and trametinib was initiated in December 2019. A decrease in size was observed at both sites, and surgery was planned (Figure 1B); however, a CT scan in March 2020 demonstrated disease progression, with enlargement of the pulmonary nodule and mediastinal and hilar lymphadenopathy. Cranial CT excluded the presence of brain metastases. Laboratory tests showed a slightly elevated LDH value (451 U/L). According to the tumor board decision, ipilimumab (IPI) and nivolumab (NIVO) combination therapy was initiated. From August to October 2020, the patient underwent four cycles of IPI (3 mg/kg of body weight) + NIVO (1 mg/kg of body weight) therapy, with no adverse events recorded.

Maintenance therapy with nivolumab was initiated after combination therapy. The patient received a total of twenty courses of nivolumab (2 cycles of 240 mg given 2 weeks apart, then 480 mg every four weeks) between November 2020 and May 2022. One month after completing four cycles of IPI + NIVO combination therapy, a CT scan demonstrated a reduction in the size of the pulmonary metastases, while the previously enlarged lymph nodes showed no radiologic change. Serial cranial CT examinations consistently excluded intracranial metastatic involvement. In July 2021, there was a notable progression of the left axillary lymphadenopathy, while all other findings remained unchanged. The patient subsequently underwent surgery, during which the primary tumor was excised with a 10 mm safety margin. The resulting skin deficit was covered with a skin graft. Histological analysis confirmed complete excision of an ulcerated nodular melanoma with partial regression, Clark level IV, mitotic activation > 1/mm^2^, and a Breslow thickness of 14 mm (Figure 1C,D).

**Figure 2:**
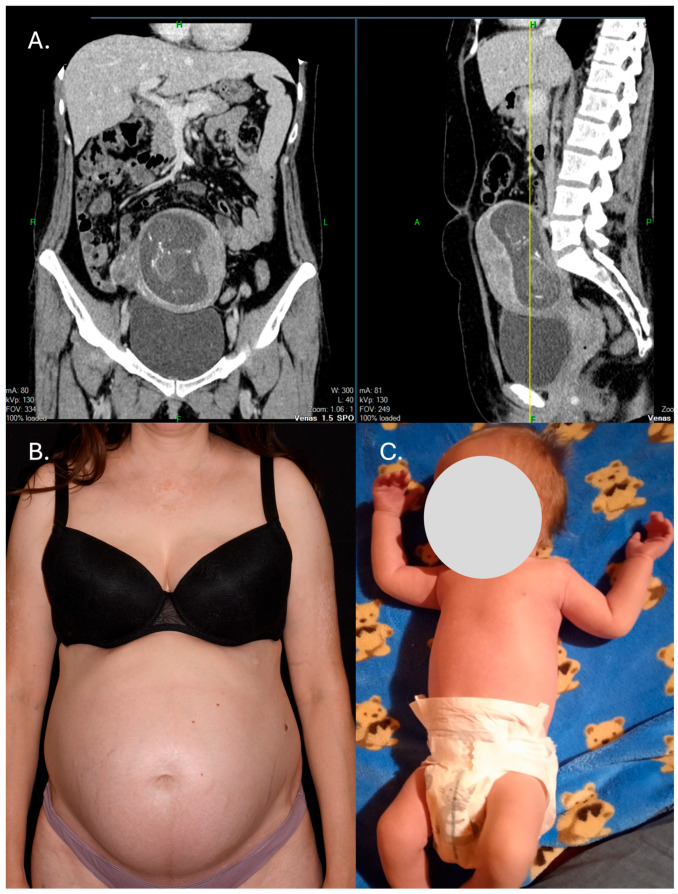
Complete regression of the pulmonary metastases and the detection of pregnancy by chance (April 2022) (**A**); 30th week of pregnancy (**B**); a healthy newborn boy delivered at 38 weeks of gestation (**C**).

In May 2022, a CT scan revealed regression of the lung metastases; however, pregnancy was detected as a secondary finding. A concurrent gynecological ultrasound confirmed a 16-week pregnancy. Genetic counseling was conducted, but the patient declined amniocentesis. A quadruple screening test indicated a low risk for congenital abnormalities. Given the patient’s favorable and sustained oncological response, the multidisciplinary team discussed the possibility of discontinuing nivolumab therapy. In parallel, the patient expressed her strong desire to continue the pregnancy. In light of both the stable disease status and the patient’s wishes, nivolumab was discontinued upon detection of the pregnancy. The patient developed gestational diabetes at the 30th week of pregnancy; besides this, the pregnancy progressed without any further complications. She gave birth to a healthy baby boy in the 38th gestational week, with a birth weight of 3120 g, a length of 49 cm, and a score of 10/10 (Figure 2).

After delivery, the patient’s oncologic status was reassessed, and no further systemic therapy was initiated due to her sustained remission and stable clinical condition. The most recent CT imaging (September 2025) and laboratory results indicate no sign of progression. The enlarged inguinal mass is still palpable, but there is no change in size (stable disease). The child continues to exhibit normal growth and development and remains in good overall health. In relation to continuous surveillance, regular physical examinations and staging CT scans are performed every 6 months, and the child undergoes age-appropriate routine pediatric follow-up.

## 3. Discussion

Pregnancy during active cancer treatment is an uncommon occurrence, representing approximately 0.1% of pregnancies and accounting for about 0.07% to 0.1% of all malignant tumors. However, although the number of cases of pregnancies during cancer treatment remains uncommon, the deliberate or unintentional administration of ICIs in pregnant patients is projected to increase. This trend is likely to accelerate following the recent approval of anti-PD-1 therapies for breast cancer, which may lead to increased unintentional exposure of pregnant individuals to ICIs [5]. Besides breast cancer, Hodgkin lymphoma, leukemia, and cervical cancer, melanoma is one of the most diagnosed cancer types during pregnancy [5,6]. It accounts for 8% of all malignant tumors diagnosed during pregnancy [7]. Managing cancer in pregnant patients presents significant challenges, as it requires careful consideration of both maternal health and fetal well-being, underscoring the need for clinically reliable data to guide treatment decisions [5].

Melanoma treatment has changed substantially in the past decade with the introduction of ICIs. These agents eliminate key negative regulators of T-cell activation, thereby enhancing antitumor immune responses, leading to extended survival and long-lasting responses in a growing number of patients [8]. The safety and efficacy of ICIs have been widely evaluated in numerous clinical trials; however, pregnant patients generally fall outside the eligibility criteria of these clinical trials. Thus, when treating this group of patients, physicians must rely on the extremely limited data available, mainly sourced from animal studies and case reports [1].

Andrikopoulou et al. published a case series of seven separate cases of pregnant patients being exposed to ICIs. In all seven cases, the underlying disease requiring treatment was malignant melanoma. Out of seven pregnancies, two were twin pregnancies [9,10]. Immunotherapy was initiated before pregnancy in four cases. Five pregnancies were exposed to immunotherapy during the first trimester, whereas two were exposed only during the second or third trimester. Two patients received four cycles of nivolumab/ipilimumab, while one patient underwent two cycles of the combination therapy, and another received only one cycle. Additionally, in one patient, four cycles of ipilimumab were administered. Only one pregnancy resulted in vaginal delivery; in the other six cases, cesarean section was performed. Placental melanoma micrometastases were reported in one case at the maternal site [11]. While metastasis to the placenta from cancers other than melanoma is uncommon, melanoma is the malignancy most frequently associated with placental metastasis [12]. Only one of the pregnancies ended in full-term birth at 38 weeks of gestation [13]. Regarding the health of the neonates, only three were born completely healthy. The remaining six neonates exhibited various conditions, including respiratory distress syndrome, intraventricular hemorrhage, retinopathy of prematurity, congenital hypothyroidism, upper limb malformation, and severe combined immunodeficiency. In five of the seven cases, adverse events occurred during pregnancy, like intrauterine growth restriction (IUGR), HELLP syndrome, placental insufficiency, and low fetal heart rate. Immune-related adverse events, including grade 1 diarrhea and immune-mediated hepatitis, occurred in two mothers.

However, none of the case reports provide information regarding the newborns’ family histories or potential genetic predispositions. Consequently, the only external factor discussed as potentially contributing to the neonatal complications is the maternal ICI exposure during pregnancy, which constitutes a limitation of these reports [12].

These clinical observations underscore the need to examine the immunological mechanisms that govern maternal–fetal tolerance, as understanding these pathways is essential for interpreting the potential risks of ICI exposure during pregnancy. Under physiological conditions, the essential role of the PD-1/PD-L1 pathway is preserving maternal immune tolerance during pregnancy, enabling the immune system to support the coexistence of two organisms, since mother and fetus are never genetically identical. For the fetus to develop successfully during gestation, the maternal immune system must regulate its response to inherited paternal alloantigens. Regulatory T-cells (Treg) play a major role in maintaining immune system balance and tolerance during pregnancy. If the PD-1 pathway is blocked by immunotherapy, this could result in an augmented fetal rejection, consequently leading to spontaneous abortion, premature deliveries, and fetal deaths, all of which were noted in animal studies, though in surviving animals, no elevated risk of congenital abnormalities was observed [1,2,12,14,15]. Checkpoint inhibitors targeting the PD-1 pathway may pose a greater risk of fetal loss or congenital abnormalities compared to anti-CTLA-4 inhibitors. This has been studied in murine models: PD-1 blockade was found to eliminate the protective role of Treg-induced fetal protection, while CTLA-4 blockade did not have this effect [13,15].

Therefore, anti-PD1 agents are classified as pregnancy category D by the Food and Drug Administration, while ipilimumab is deemed safer and assigned pregnancy category C due to the absence of preclinical evidence supporting a critical role for the CTLA-4 axis in fetal-immune tolerance [14].

In preclinical studies of nivolumab, no teratogenic effect could be shown in surviving progenies, but some congenital abnormalities occurred with ipilimumab [2].

In animal studies, when ipilimumab was given to cynomolgus monkeys, a dose-dependent increase in the incidence of third-trimester miscarriage, stillbirth, premature delivery, low birth weight, and infant mortality could be observed. Adverse effects could not be detected in the first two trimesters. A key limitation of the available animal studies is the absence of long-term follow-up data on offspring exposed to ICIs in utero [11,13].

Another concern with administering ICIs during pregnancy is the frequent occurrence of immune-related adverse events, such as immune-related hypophysitis, which could lead to abnormalities in pituitary hormone production, resulting in impaired secretion of follicle-stimulating hormone and luteinizing hormone. Comparing the two main agents when treating melanoma with ICIs, anti-CTLA-4 inhibitors are more likely to cause hypophysitis than PD-L1 inhibitors. Fetal outcome could also be negatively affected if, in case of occurrence of the above-mentioned side effect, corticosteroids were administered [12].

Anti-PD-1 agents such as nivolumab or pembrolizumab are immunoglobulin G4 (IgG4) antibodies that have the potential to cross the placenta and cause immune-related adverse events in the fetus. Fetal IgG levels are low in the first two trimesters but increase gradually in the third trimester, reaching concentrations comparable to those in the maternal bloodstream. Accordingly, nivolumab and pembrolizumab can be transferred from the mother to the fetus, potentially triggering immune-related effects/autoimmune events such as congenital hypothyroidism stemming from an immune-related thyroiditis in the fetus. Therefore, it can be stated that the risk of immune-mediated side effects is more likely in the third trimester rather than during organogenesis because of the increased level of IgG4 antibodies in the fetus. Given the low exposure to maternal antibodies during organogenesis, patients should not be encouraged to abort when conceiving under ICI treatment [12,15]. Recently, Baarslag et al. published a case report describing a 4-month-old infant who developed severe immune-related enteritis following in utero exposure to pembrolizumab. The case is significant because, despite being born healthy, the infant later experienced immune-mediated gastrointestinal toxicity as a consequence of prenatal PD-1 inhibitor exposure. This case highlights the possibility of delayed immunotoxicity and further emphasizes the need for cautious interpretation of outcomes and close postnatal monitoring [16].

In pregnant patients diagnosed with cancer, it is crucial to understand the obstetric and neonatal risks linked to both the disease and its treatment options. Efforts to prevent preterm delivery by managing cancer during pregnancy must be carefully balanced against the increased risk of small-for-gestational-age infants. While the short-term and long-term consequences of this growth restriction are significant, the potential complications associated with preterm birth also warrant careful consideration. Further long-term studies are needed to compare the risks faced by these two groups and to inform optimal management strategies [6].

According to Rzeniewicz et al., there is evidence indicating that gestation extending beyond 36 weeks may elevate the risk of transplacental melanoma transmission; elective delivery between 34 and 36 weeks should be considered for patients diagnosed with gestational melanoma, followed by appropriate systemic therapy [2].

Gougis et al. conducted a prospective study using the WHO’s VigiBase to explore pregnancy outcomes linked to anticancer therapies, including ICIs. Among 3558 reported cases of anticancer drug use during pregnancy, 91 involved ICIs. Adverse outcomes occurred in 41.8% of ICI-exposed pregnancies, compared to 57.1% with non-ICI drugs. Notably, no specific pregnancy, fetal, or neonatal complications were significantly overreported in the ICI group. Combination ICI therapy (PD-1 and CTLA-4 inhibitors) was linked to a higher risk of preterm birth compared to non-ICI anticancer treatments, a risk not seen with ICI monotherapy. Additionally, three cases in the ICI group reported possible immune-related complications [17].

In summary, it can be stated that according to the limited data available, the most commonly occurring side effects of ICIs in case of in utero exposure are fetal growth restriction, premature delivery, fetal distress syndrome, and, in a few cases, congenital abnormalities such as congenital hypothyroidism or congenital hand malformation. During follow-up, several infants were reported to be in good health and progressing appropriately in their developmental milestones, although follow-up data were limited and not available for all cases [2,12].

## 4. Conclusions

The increasing use of ICIs such as PD-1 and CTLA-4 inhibitors has significantly advanced melanoma treatment. However, their administration during pregnancy remains complex and controversial due to limited data and potential risks to the fetus. In our case, a young woman treated with maintenance nivolumab therapy for metastatic melanoma was incidentally found to be pregnant. She delivered a healthy male infant at 38 weeks with no significant complications, highlighting that favorable outcomes are possible with careful management. In the case of our patient, following the diagnosis of pregnancy at 16 weeks of gestation, maintenance nivolumab therapy was discontinued due to the good clinical response, and the patient elected to continue the pregnancy. As treatment was stopped during the second trimester, no immune-mediated adverse events were observed. Furthermore, nivolumab exposure did not result in any teratogenic effects on the fetus. These observations are congruent with the limited data that are available on the effect of ICIs on pregnancy.

Current evidence suggests that in utero exposure to ICIs may be associated with adverse fetal outcomes such as growth restriction, premature delivery, fetal distress, and congenital anomalies like hypothyroidism and limb malformations. Nonetheless, follow-up in reported cases indicates that many infants develop normally. Animal studies further suggest that PD-1 blockade could impair maternal-fetal immune tolerance, potentially leading to pregnancy loss or fetal abnormalities, especially during the third trimester when maternal IgG transfer increases.

Given the paucity of data—only a handful of case reports exist—there is an urgent need for further long-term studies to establish guidelines. Treatment decisions should involve individualized risk assessments and a multidisciplinary team to balance maternal benefits against fetal risks. Our case contributes to the limited but growing evidence supporting the possibility of successful pregnancy outcomes during ICI therapy in melanoma patients.

However, this case report also has some limitations that must not be overlooked: our case report only describes a single case. In addition, long-term follow-up of the patient and the child must be conducted. By acknowledging these limitations, we aim to provide a balanced perspective while emphasizing the relevance of our findings within the scarce existing literature.

The decision to initiate treatment during pregnancy should consider factors such as the urgency of treatment, anticipated maternal benefits, and the gestational age at the time of exposure. Furthermore, the importance of contraception should be emphasized for all patients of childbearing potential undergoing cancer treatment.

## Data Availability

No new data were created or analyzed in this study. Data sharing is not applicable to this article.

## Abbreviations

The following abbreviations are used in this manuscript:

ICIs	Immune checkpoint inhibitors
PD1	Programmed cell death 1
PD-L1	Programmed cell death ligand 1
CTLA- 4	Cytotoxic T lymphocyte antigen 4
IUGR	Intrauterine growth restriction
Treg	Regulatory T-cells
IgG4	Immunoglobulin G4
